# Automatic grading evaluation of winter wheat lodging based on deep learning

**DOI:** 10.3389/fpls.2024.1284861

**Published:** 2024-04-25

**Authors:** Hecang Zang, Xinqi Su, Yanjing Wang, Guoqiang Li, Jie Zhang, Guoqing Zheng, Weiguo Hu, Hualei Shen

**Affiliations:** ^1^ Institute of Agricultural Information Technology, Henan Academy of Agricultural Sciences, Zhengzhou, China; ^2^ Huanghuaihai Key Laboratory of Intelligent Agricultural Technology, Ministry of Agriculture and Rural Areas, Zhengzhou, China; ^3^ College of Computer and Information Engineering, Henan Normal University, Xinxiang, China; ^4^ School of Life Science, Zhengzhou Normal University, Zhengzhou, China; ^5^ Wheat Research Institution, Henan Academy of Agricultural Sciences, Zhengzhou, China

**Keywords:** UAV image, winter wheat, deep learning, lodging degree, lodging area

## Abstract

Lodging is a crucial factor that limits wheat yield and quality in wheat breeding. Therefore, accurate and timely determination of winter wheat lodging grading is of great practical importance for agricultural insurance companies to assess agricultural losses and good seed selection. However, using artificial fields to investigate the inclination angle and lodging area of winter wheat lodging in actual production is time-consuming, laborious, subjective, and unreliable in measuring results. This study addresses these issues by designing a classification-semantic segmentation multitasking neural network model MLP_U-Net, which can accurately estimate the inclination angle and lodging area of winter wheat lodging. This model can also comprehensively, qualitatively, and quantitatively evaluate the grading of winter wheat lodging. The model is based on U-Net architecture and improves the shift MLP module structure to achieve network refinement and segmentation for complex tasks. The model utilizes a common encoder to enhance its robustness, improve classification accuracy, and strengthen the segmentation network, considering the correlation between lodging degree and lodging area parameters. This study used 82 winter wheat varieties sourced from the regional experiment of national winter wheat in the Huang-Huai-Hai southern area of the water land group at the Henan Modern Agriculture Research and Development Base. The base is located in Xinxiang City, Henan Province. Winter wheat lodging images were collected using the unmanned aerial vehicle (UAV) remote sensing platform. Based on these images, winter wheat lodging datasets were created using different time sequences and different UAV flight heights. These datasets aid in segmenting and classifying winter wheat lodging degrees and areas. The results show that MLP_U-Net has demonstrated superior detection performance in a small sample dataset. The accuracies of winter wheat lodging degree and lodging area grading were 96.1% and 92.2%, respectively, when the UAV flight height was 30 m. For a UAV flight height of 50 m, the accuracies of winter wheat lodging degree and lodging area grading were 84.1% and 84.7%, respectively. These findings indicate that MLP_U-Net is highly robust and efficient in accurately completing the winter wheat lodging-grading task. This valuable insight provides technical references for UAV remote sensing of winter wheat disaster severity and the assessment of losses.

## Introduction

Wheat is a food crop worldwide, providing sustenance for approximately a third of the global population (Zhang et al., 2014; Wen et al., 2022). According to the Food and Agriculture Organization, the global wheat planting area was 223 million hectares, yielding 776 million tons (FAO, 2021). Given China’s increasing population and decreasing arable land, boosting wheat yields is the key to achieving self-sufficiency. However, wheat production is frequently hampered by extreme weather, such as typhoons, heavy rains, and hailstorms, bringing many uncertainties to the wheat yield. Therefore, using unmanned aerial vehicle (UAV) remote sensing to monitor wheat disasters and predict yields has become essential to ensure food security.

Lodging is a significant problem in field production, which can cause a severe reduction in crop yields by up to 50% (Foulkes et al., 2010; Berry and Spink, 2012; Peng et al., 2014; Singh et al., 2019; Zhao et al., 2020). This phenomenon occurs when the aboveground stems lose their upright state (stem lodging) or when root soil attachment is damaged (root lodging) (Pinthus, 1974). It is commonly observed that the middle and later stages of wheat growth can cause partial or majority wheat lodging (Sara et al., 2019). Wheat lodging has been found to impact its individual development and overall yield and quality (Liu et al., 2014; Yang et al., 2021). Therefore, it is crucial to evaluate the degree and area of lodging promptly and accurately. This information is essential for analyzing wheat disasters and providing a reference for identifying the level of wheat lodging disasters and breeding improved varieties. Furthermore, it is a critical basis for agricultural insurance companies to assess the degree of wheat lodging and determine agrarian losses.

In crop breeding practice, researchers are working on developing fall-resistant wheat varieties (Piñera-Chavez et al., 2016) and prediction models for extreme weather events (Sterling et al., 2003). One of the challenges in this field is acquiring wheat lodging information, typically done through manual and remote sensing measurements. However, these methods can be subjective and time-consuming. Therefore, remote sensing measurement has been developed based on spectral, texture, and color characteristics to monitor wheat lodging information in different regions. Chauhan et al. (2020) developed a multitemporal discriminant analysis method that uses partial least squares to classify the severity of wheat lodging. Chauhan et al. (2019) have analyzed the spectral variability of different lodging severity levels using UAV multispectral data. They have also classified them using high-resolution UAV data. Zhang et al. (2020) have employed a UAV system for image acquisition and machine learning algorithms to detect the occurrence of wheat lodging. Tian et al. (2021) utilized multispectral and RGB cameras installed on a UAV platform to analyze the image features of non-lodging and lodging rice. They have examined several factors, such as spectral reflectance, vegetation index, texture, and color, to optimize lodging detection indicators. After analyzing these factors, they established a rice lodging detection model based on selected image features to distinguish between non-lodging and lodging rice. Yang et al. (2017) proposed a hybrid spatial and spectral-based image classification technique to detect lodging areas effectively. Shu et al. (2020) presented a method that relied on changes in maize plant height to monitor the degree of lodging, using dual-polarization Sentinel-1A data to calculate the lodging angle. Sun et al. (2019) employed maximum likelihood classification to classify the UAV multispectral image features and extract four maize lodging grades.

Artificial intelligence (AI) has significantly increased agricultural information in recent years. Various studies have applied AI techniques, such as machine vision, to this area with promising results (Kamilaris and Prenafeta-Boldú, 2018; Bu and Wang, 2019; Liu et al., 2019; Nguyen et al., 2020; Song et al., 2020; Yang et al., 2020; Koh et al., 2021; Mao et al., 2022). Wilke et al. (2019) used SfM technology to quantify barley’s lodging area and severity. Rajapaksa et al. (2018) extracted texture features from wheat UAV images using a gray-level co-occurrence matrix, local binary patterns, and a Gabor filter to classify lodging degrees. Studies have also explored various methods for analyzing and predicting the lodging area in crops, using deep learning and neural networks. Su et al. (2022) used an improved U-Net network to statistically analyze lodging wheat through small-sample training. Yang et al. (2020) combined edge computing with EDANet to predict lodging areas quickly and effectively. Zhao et al. (2019) proposed a new method for evaluating rice lodging based on a deep learning U-Net structure, facilitating efficient extraction of rice lodging areas in a large area. Zhang et al. (2020) proposed a method based on transfer learning and the DeepLabv3+ network to extract lodging areas in different growth wheat stages, which is better than the traditional U-Net. Tang et al. (2022) proposed a semantic segmentation network model called PTCNet, which performed well on high-resolution satellite datasets by integrating features from multiple scales.

However, the above researchers mainly adopted conventional machine and deep learning methods for feature classification. They have failed to combine and optimize different feature screening and classification methods. As a result, the accuracy of the results is low, and their results lack universality. Deep learning algorithms in this study mainly use raw images that have not been spliced to extract the lodging area. However, this area has high heading and lateral overlap, which requires manual deduplication before and after image processing. Moreover, none of the abovementioned methods analyzed the degree and area of lodging combined. This study aims to construct and label lodging datasets for different wheat varieties and improve the technique of extracting lodging for plot areas with different regions and flight heights. Additionally, the study seeks to establish a classification semantic segmentation dual task neural network model MLP_U-Net while completing the classification of lodging degree and lodging area categories and establishing a joint weighted loss balance for multiple task weights to prevent gradient explosion.

## Materials and methods

### Study area

**Figure 1:**
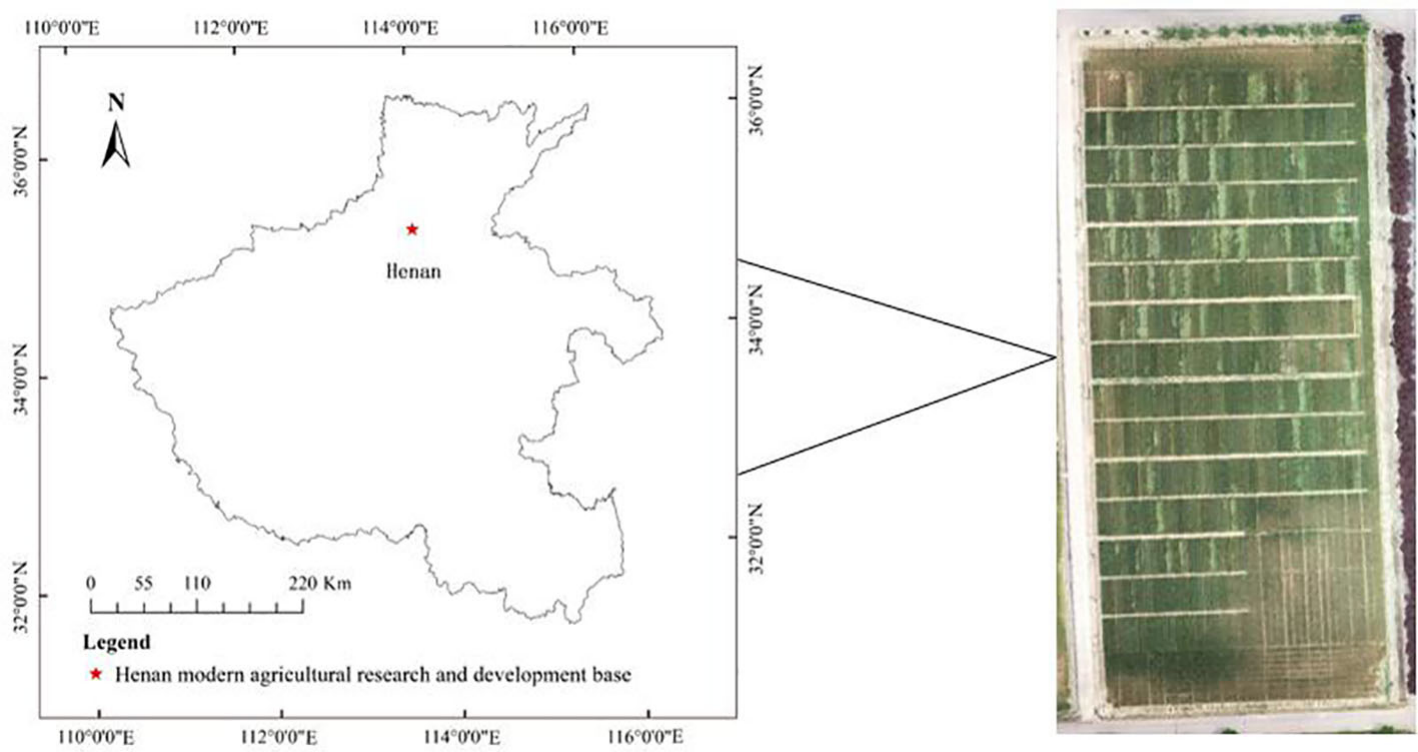
Location of the study area.

The study area is located in the winter wheat regional experiment at Henan Modern Agricultural Research and Development Base of Henan Academy of Agricultural Sciences. According to the details provided, this study area is located at 35°0′44″ north latitude and 113°41′44″ east longitude, with an altitude of 97 m, as shown in **Figure 1**. The region of Yuanyang County, located in the North China Plain, falls under a warm temperate continental monsoon climate, with the primary crop grown in autumn being winter wheat. The average annual rainfall and temperature are 549.9 mm and 14.4°C, respectively, with annual sunshine hours ranging from 2,300 h to 2,600 h. Winter wheat in this study area is in the filling period, and the region is likely to experience a high risk of lodging due to the extreme climate.

A total of 82 winter wheat varieties were tested in a completely randomized group design with three replications and a plot area of 12 m^2^. The seeds were sown during the appropriate sowing period according to the experimental plan, and the field management measure is higher than those of the ordinary field.

### Data collection

This study used the DJI 4 Pro UAV with a wheelbase of 350 mm, camera pixels of 20 million pixels, image sensor of 1 inch CMOS, lens parameters of FOV 84°, 8.8 mm/24 mm (35-mm format equivalent), and aperture of f/2.8-f/11. Equipped with GPS/GLONASS dual-mode positioning, the captured image resolution is 5,472 pixels × 3,078 pixels, and the aspect ratio is 16:9. The flight adopts the route automatically planned by the DJI UAV, and the aerial photography is completed and landed with automatic return. The image collection time for flyover 1 was 10:00 am on 14/05/2020, the weather clear and cloudless, and it was taken vertically, with the flight altitude of 30 m, flight speed of 3 m/s, and flight duration of 25 min; the heading overlap and lateral overlap were both 80%, camera photography mode was taken at equal time intervals, and 700 original images were finally collected. The hardware facilities and parameter settings of flyover 2 are the same as flyover 1, with the collection time of 20/05/2022 and the flight altitude of 50 m. Regardless of the changes in flight altitude between flyover 1 and flyover 2, as long as the flight altitude is controlled in a controlled operating environment, through appropriate training and parameter adjustments, this research method has a certain degree of universality and accuracy.

### Data preprocessing

The collected data must be preprocessed, and the PIX4D mapper software is used for radiometric calibration and geometric correction on the original image. This process is done to obtain digital orthophoto images of the experimental field. Once the images are concatenated, the resolution is 5,153 pixels × 3,999 pixels. The stitched image is cropped and appropriately rotated, and the processed image resolution becomes 1,196 pixels × 2,853 pixels, as shown in **Table 1**.

**Table 1:** Dataset processing.

Dataset processing	Flyover 1	Flyover 2
Image size of dataset	1196 pixels×2853 pixels	1196 pixels×2853 pixels
samples number of original dataset (image)	1	1
Preprocessed image size for each plot	128 pixels×512 pixels	128 pixels×512 pixels
Number of plots after pre-treatment (image)	487	487
Number of training set (image)	689	689
Number of testing set (image)	159	131

**Figure 2:**
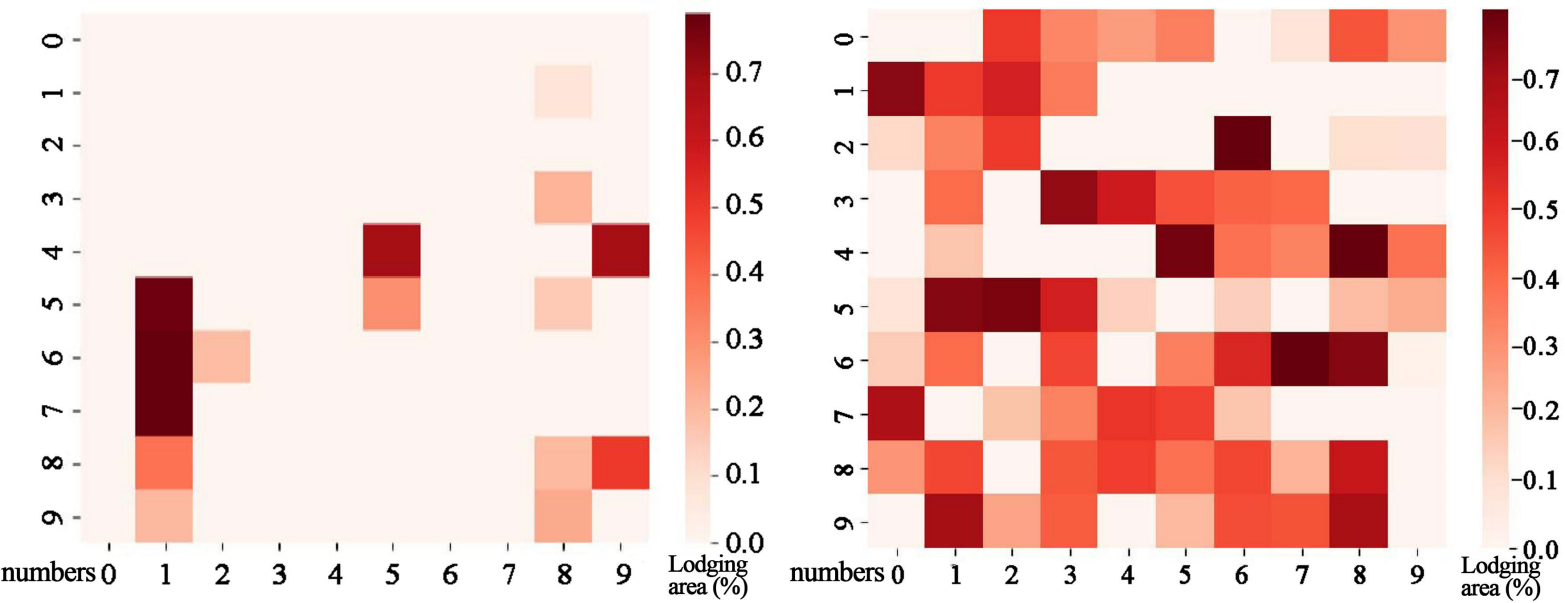
Data sample distribution.

As shown in **Figure 2**, the darker the color, the higher the degree of lodging. Additionally, the number of lodging plots in flyover 2 is more than that in flyover 1. Additionally, there is a significant difference in training difficulty due to the varied distribution of data samples in different datasets. Therefore, flyover 1 has fewer lodging plots than flyover 2, making training less complicated. Moreover, flyover 1 is undersampled, and flyover 2 is oversampled. The two flyovers were located in the same experimental field in different years, covering 15 rows. Flyover 1 covered 487 plots of 82 winter wheat varieties with uniform data distribution. However, linear transformation for data enhancement may result in uneven data distribution. This study collected samples from flyover 2 in the same region in 2022 to increase the sample size. Flyover 1 uses nine rows of data from the north of the study area for the training set, while flyover 2 uses 10 rows from the north for the training set. Other data are used as the test set. The specific process comprises the following five steps:

1. Classification data labeling: A labeling tool was used to annotate the plots as VOC format data. Flyover 1 used integrated field sampling and visual interpretation, and flyover 2 used visual interpretation.
2. Cell extraction: This step is conducted using RoIAlign (Chauhan et al., 2020) to generate candidate regions in the original data according to the location coordinates of the labeled data using the bilinear interpolation method. Then, the candidate regions are mapped to produce a feature map that is 128 pixels × 512 pixels in size.
3. Manual labeling: Semantic segmentation pixel-level labeling of images was done using the LabelMe tool. The lodging wheat region was marked as the foreground, and the non-lodging wheat region was marked as the background, which was converted to a binary image label.
4. The classification part of the model generates the prediction category, while the segmentation part generates the mask map. Then, the mask map is mapped with ground object relationships to obtain the predicted area value of the plot. From there, the percentage of the lodging area in the plot’s total area is calculated to obtain the lodging area prediction category. The predicted data are downsampled to the original resolution and overlaid onto the source data graph by labeling the data position, making the predicted results clear and visible.
5. Accuracy verification: The model evaluation index is calculated using the mask map generated by the model, and the model prediction’s accuracy is evaluated by comparing it with the labeled data through the confusion matrix. This method helps verify the model’s accuracy and determine its performance in predicting the expected outcomes.

**Figure 3:**
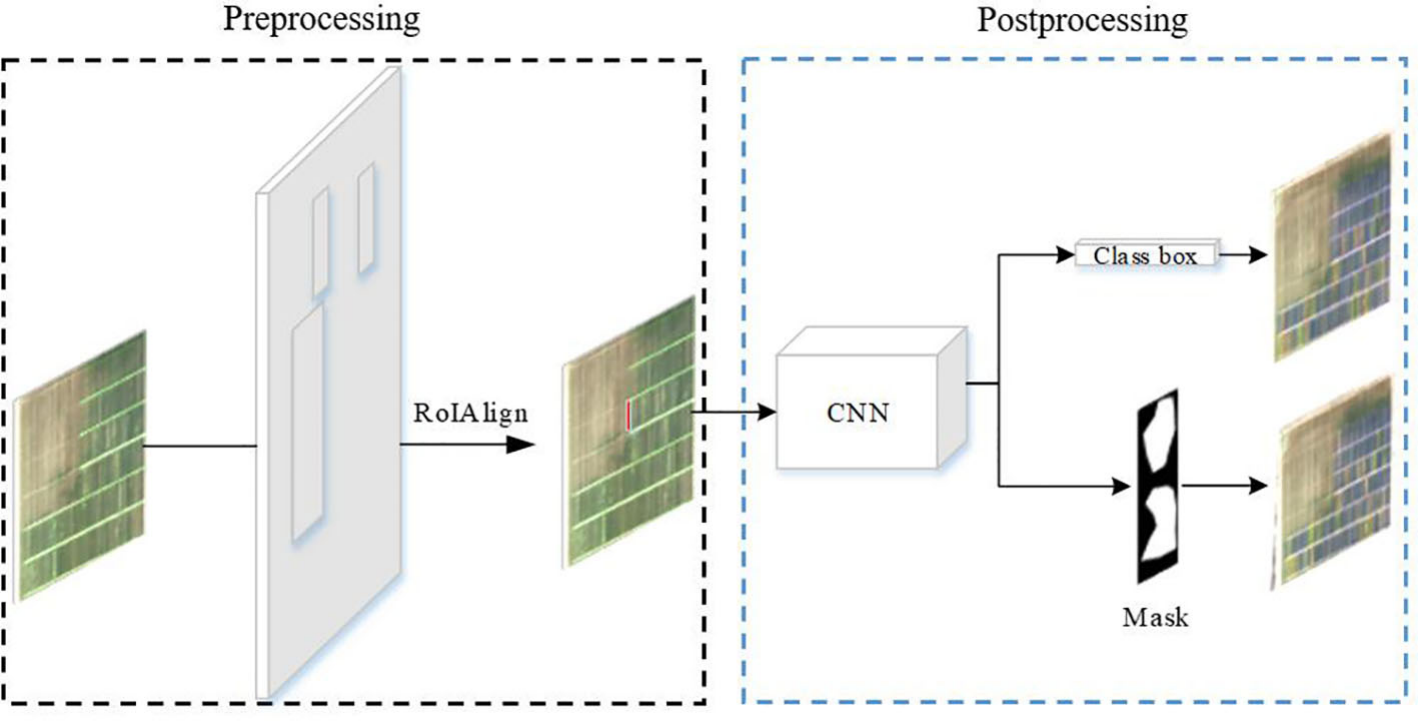
Data preprocessing and postprocessing.

**Figure 4:**
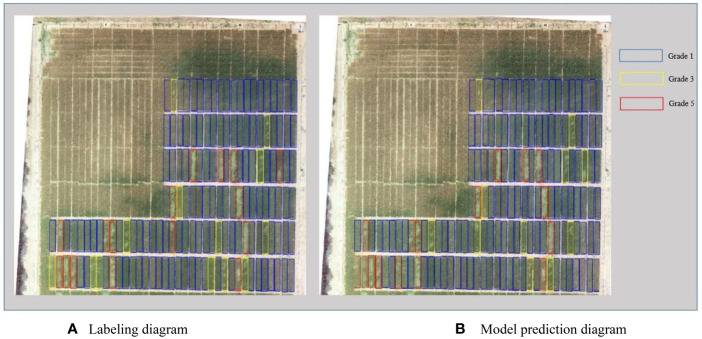
Qualitative analysis of lodging degree grading for dataset 1. **(A)** Labeling diagram; **(B)** Model prediction diagram.

**Figure 5:**
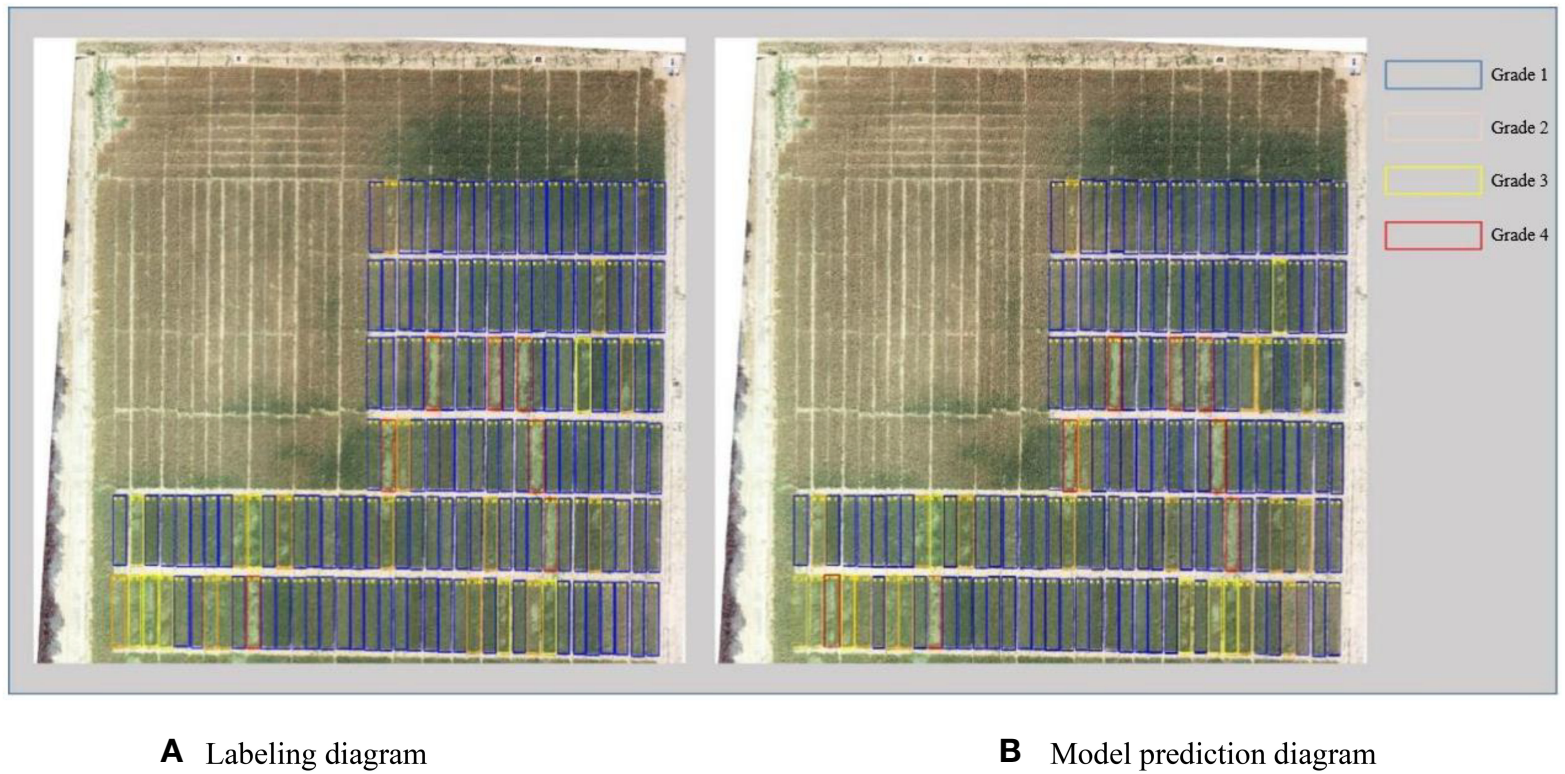
Qualitative analysis of lodging area grading for dataset 1. **(A)** Labeling diagram; **(B)** Model prediction diagram.

**Figure 6:**
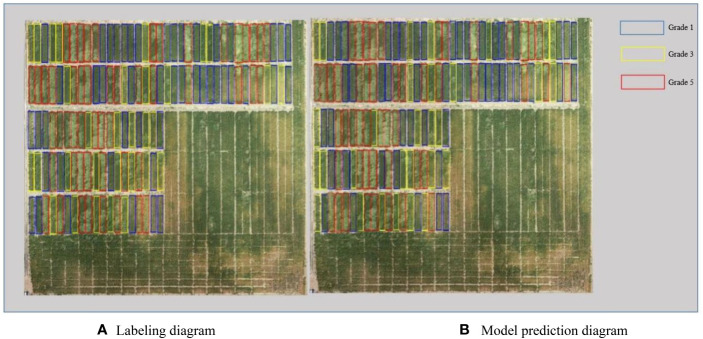
Qualitative analysis of lodging degree grading for dataset 2. **(A)** Labeling diagram; **(B)** Model prediction diagram.

**Figure 7:**
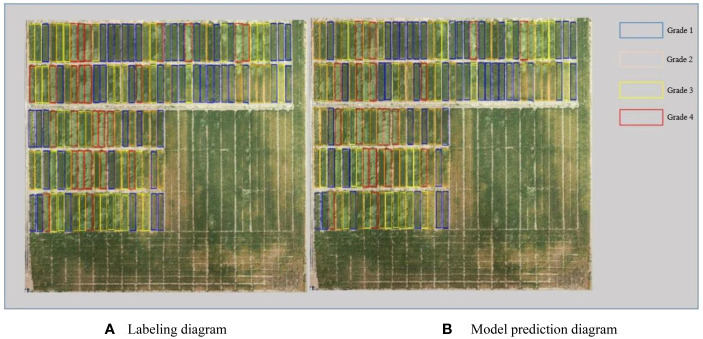
Qualitative analysis of lodging area grading for dataset 2. **(A)** Labeling diagram; **(B)** Model prediction diagram.

As shown in **Figure 3**, the source data are input to the neural network through RoIAlign to generate candidate regions for each plot. The neural network outputs prediction classification and a mask map. The predicted lodging area values are obtained from the mask map. These values are then combined with the source data to obtain the lodging degree grading (**Figures 4**, **5**) and the lodging area grading (**Figures 6**, **7**) of the prediction map.

However, the resolution of a single image after stitching processing is quite large, measuring 1,196 pixels × 2,853 pixels, posing challenges for deep learning to process the stitched image directly. Therefore, training and testing the data before stitching may be more practical, as this can help avoid manual deduplication of highly overlapping data. As a result, we designed a multitask neural network model training to reduce the overfitting problem by training the model with small samples to achieve good results. Moreover, we set randomly discarded neurons in the multitask neural network to disturb and add noise between different tasks using multitask learning to improve the network’s robustness.

### Multitask learning model

Our model is developed based on the multitask learning method (Caruana, 1993; Zhang et al., 2014; Ranjan et al., 2017; Ruder, 2017; Zhang and Yang, 2018), which differs from single-task learning. Most conventional machine learning models are single-task learning, such as lodging region segmentation and wheat spike counting. However, multitask learning involves solving simple and independent subproblems individually and combining their results to obtain the results of a complex problem. This approach is simple to implement but has some limitations. Each subproblem is not independent, and they may be correlated. If the real problem is divided into multiple subproblems, the rich correlation information between the issues will be ignored, and multitasking learning will be created to solve this problem. Using a shared layer between multiple tasks, a multitask learning model can leverage their correlation and improve learning. However, some information may not be as relevant to all functions, including low correlation between tasks, which can instead bring random noise and hinder the ability to achieve better generalization results.

### MLP_U-Net model structure

The encoder–decoder framework is commonly used for semantic segmentation tasks by cascading the encoder and decoder information. This model is effective in recovering fine-grained details in complex backgrounds. U-Net is a popular model that uses this framework in medical image processing, and its performance is excellent. Moreover, this model has become a mainstream semantic segmentation model. However, the depth of the encoder and decoder networks must be continuously adjusted to achieve the best results according to the task difficulty and the amount of labeled data available for training. UNet++ indirectly fuses several different levels of features through short joins and up- and downsampling operations (Zhou et al., 2018). However, merging features at the same level of the encoder and decoder in UNet++ is beneficial in allowing the encoder to process features with various sensory field sizes. This approach also enables the network to meet the demands of different data volumes and tasks for network depth. Despite these advantages, UNet++ still has many parameters and limited extraction capability for multiscale features.

Multilayer perceptron (MLP) is a feedforward artificial neural network model consisting of input, hidden, and output layers, with deep layers and multiple nodes in each layer (Pinkus, 1999). Multilayer perceptron layers are interconnected, and its perceptron can contain multiple hidden layers. In recent years, the success of models based on MLPs has proven that neither convolutional nor attention mechanisms are necessary conditions for excellent model performance. MLP-Mixer is a model based on a multilayer perceptron, replacing the convolutional operation in traditional CNN and the self-attention mechanism in Transformer (Llya et al., 2021). This model divides the input image into several patches and simultaneously maps the rows and columns, allowing for better information fusion in the channel and spatial domains. Additionally, it can handle multiscale features well. As a result, this study designs a model based on MLP and a multitask learning method for wheat lodging grading tasks. This model is designed to complete the lodging grading task with less data volume and computational resources while having strong multiscale feature extraction capability.

**Figure 8:**
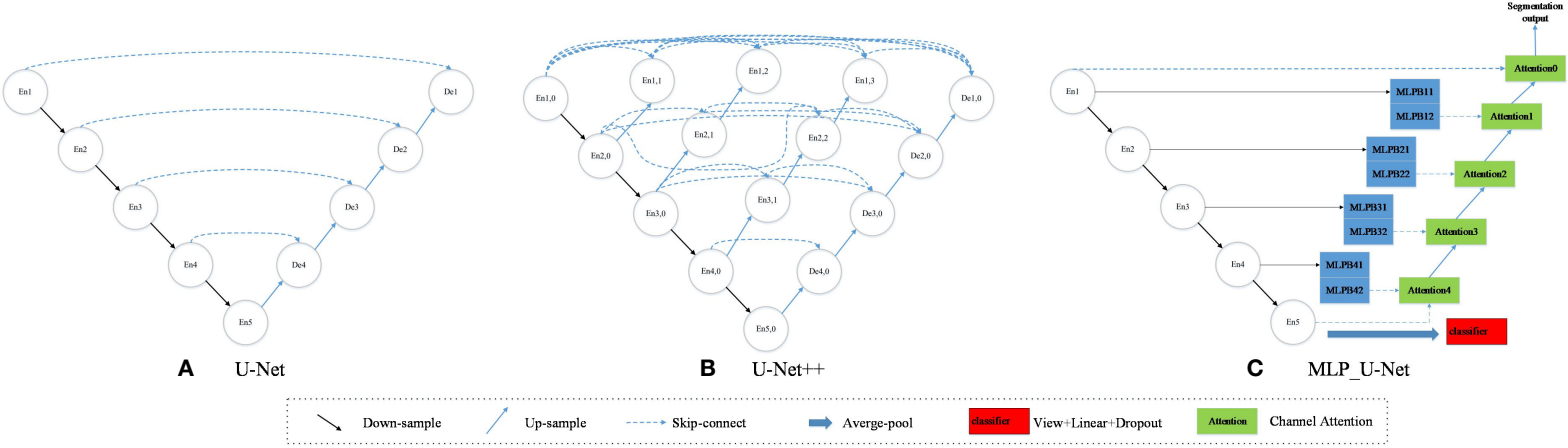
Comparison of model structure. **(A)** U-Net; **(B)** U-Net++; **(C)** MLP_U-Net.

As shown in **Figure 8**, the semantic segmentation of this model consists of a continuous downsampling part, a feature refinement part based on the MLP layer, and an upsampling part based on the channel attention mechanism. The downsampling part comprises a convolutional layer, a pooling layer, and an activation function. This study sets the number of channels in each layer to 16, 32, 64, 128, and 256 in downsampling, and the size of the feature map decreases from [512,128] to [16,4]. This simple structure and fewer parameters can significantly reduce the overfitting problem caused by small samples.

By generating a predictive classification from the downsampled feature maps after the global pooling layer, the classifier can ensure the number of common layers while keeping the task simpler than semantic segmentation. This approach may introduce some noise to the semantic segmentation task, but it also facilitates the model’s generalization.

Based on the MLP layer, our feature refinement part is structured with the same input and output size. As shown in **Figure 8**, En1–En5 represent five downsampling cycles and 1–5 represent the depth of the model. Moreover, each model layer consists of two MLP Blocks, and MLPB11–MLPB42 represent the depth of the model. Taking the bottom layer as an example, MLP Block 41 and MLP Block 42 have two consecutive Shift MLP layers. The first MLP Block uses convolution with a step size 2 to increase the input channel from C to 3/2C. Even though the model layers are deepened by En4, they have not reached the depth of En5. Therefore, we redefine this model as MLP Block41, which differs from En5. As a result, the second MLP Block uses convolution with a step size of 1 to increase the number of channels from C to 2C. We define this as MLP Block42, in which the generated feature map is the same size as the lower layer module undergoing convolution operation and exhibiting strong pluggability and scalability.

**Figure 9:**
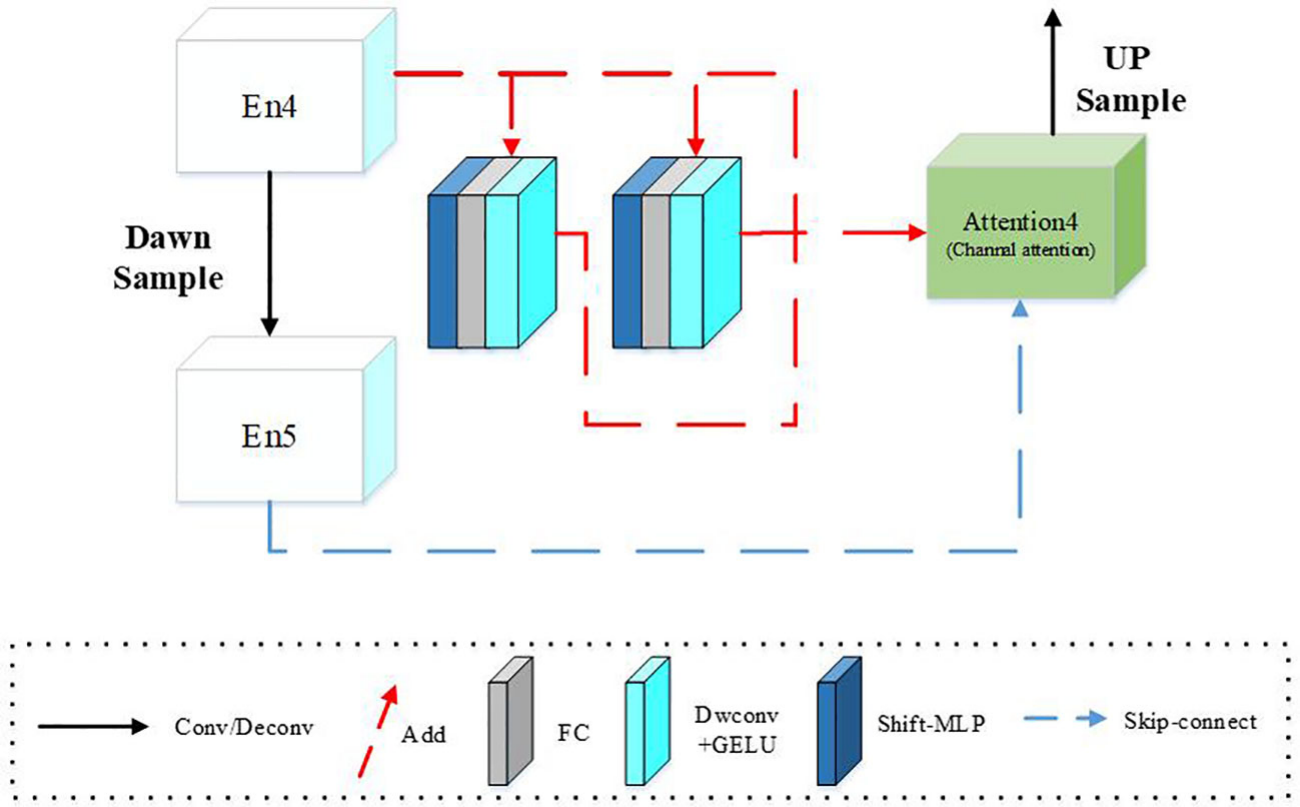
MLP_U-Net underlying model structure.

The MLP layer is depicted in **Figure 9**, where we pass the input part through two different Shift MLP layers. We also connect the residuals through a fully connected layer, DW convolution, after the activation function and the input features. DW convolution is preferred due to its fewer parameters and its efficiency. Additionally, we use GELU instead of RELU because it is a smoother alternative and performs better.

The channel attention mechanism is used to refine and splice the upsampling feature map generated by the MLP Block step by step, and upsampling is performed. This process helps to maintain the integrity of semantic information by cascading the features of the upper layer with the same resolution features of the same layer.

### Improved shift MLP module architecture

MLP has been widely used for computer vision tasks in recent years. As a result, several studies (Borghi et al., 2021; Rao et al., 2021; D'orazio et al., 2022; Touvron et al., 2022) have demonstrated superior performances of the MLP-based approaches, which do not rely on attention mechanisms. MLP-Mixer (Llya et al., 2021) is a new architecture for a model based on multilayer perceptron, which replaces the convolution operation in traditional CNN and the self-attention mechanism in Transformer (Wu et al., 2021). This MLP-Mixer divides the input image into several patches to map rows and columns simultaneously, realizing information fusion in channel and spatial domains. Spatial shift MLP (Yu et al., 2021) replaces the token-mixing in MLP-Mixer with a spatial shift operation for enhancing the connection between various patches.

**Figure 10:**
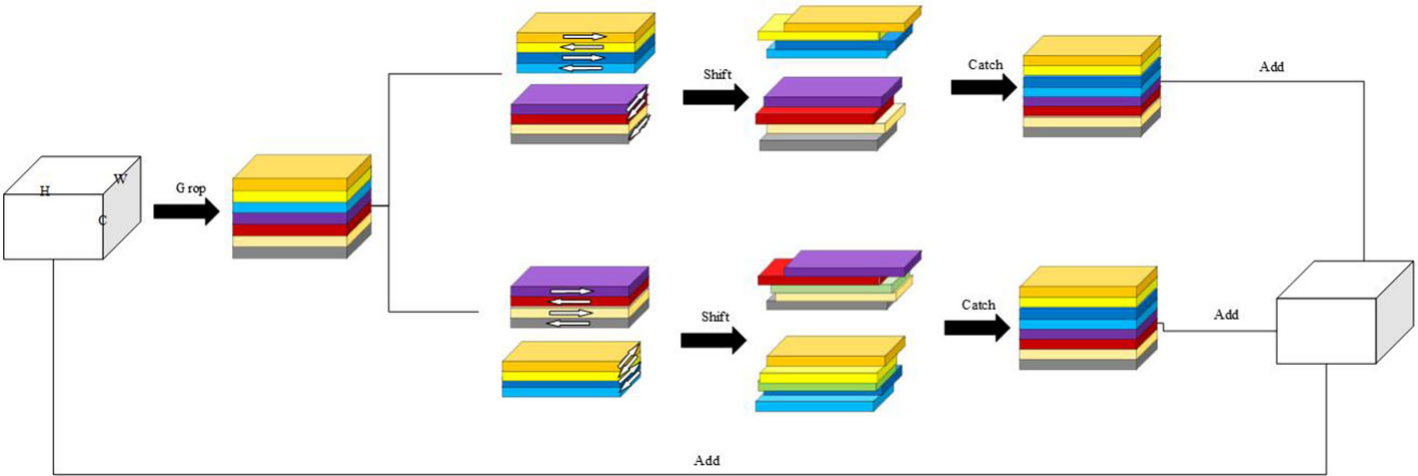
Improved Shift MLP module structure.

**Figure 10** shows an improvement in the Shift MLP architecture, where the input is divided into eight different groups, with each of the four groups offset along different axes (H-axis and W-axis). Moreover, the grouping is offset in reverse along different axes, and two blocks are pieced together. Then, we performed the residual connection between the two pieced feature maps and the input features to obtain the final feature map. When grouping is performed, the groupings cannot be completely distinguished due to the different feature map sizes. As a result, the number of channels in the last group differs from other groupings. The improved shift MLP technique involves splicing the first five groups with the previous five groups after rotating them by different axes in two. This process allows the features to be restored to the original feature map size. The purpose of setting up two axes is to ensure that each patch goes through two different shift axes in each training round. The step size of each shift can be either [−2,−1,1,2] or [2,1,−1,2]. By fusing features and shifting dimensions, combined with different semantic information in different groups, an approximate long-distance interaction process can be achieved even if only adjacent patches are associated from the perspective of a single spatial offset module based on the overall stacked structure.

### Loss of multitask learning

The loss of multitask learning has always been a major difficulty in model construction. Reasonable loss can add appropriate noise between multiple tasks and thus improve the robustness of the model; conversely, the tasks contradict each other and lead to model failure to converge. This model needs to balance the classification loss and segmentation loss. Since the semantic segmentation task is more difficult to train than the classification task, we weight the loss of each task according to the percentage between the training rounds and the loss to obtain the final loss as shown in Equation 1.


(1) $$l_{tol}=avg\left(\left(e^{-\left(\frac{l_{cls}}{l_{cls}+l_{seg}}\right)\times \alpha }+eps\times \beta \right)l_{cls}+l_{seg}e^{-\left(\frac{l_{cls}}{l_{cls}+l_{seg}}\right)\times \alpha }\right)$$

Where $l_{cls}$ is the classification loss value, $l_{seg}$ is the semantic segmentation loss value, and α and β are the custom parameters.

This study uses cross entropy and binary cross entropy to calculate the classification loss and semantic segmentation loss of the model, respectively. The parameter α is used to balance the mixing factor of the network in the final loss contribution. If the loss function of one task is far greater than that of another task, which leads to the inability of another task to learn or the gradient explosion, the task with a large proportion of loss function will be punished; otherwise, it will be weighted forward. In this study, the segmentation loss is the average of all pixels, and the misclassified pixels is smaller than the total pixels, resulting in a segmentation loss value that may be smaller than the classification loss value. After with reasonable weighting, the semantic segmentation task can be better performed without affecting the classification task.

Before the segmentation network is stabilized, it is difficult to train the classification network, and although the loss share between tasks is balanced by the parameter α, the fluctuation of the classification task at the initial stage still poses a considerable challenge to the segmentation task. Therefore, this study focuses on weighting during the training period, with a focus on training semantic segmentation tasks in the early stages. After the semantic segmentation loss gradually stabilizes, the classification loss weight is gradually increased with the training rounds.

Using F1 value as the basis for segmentation loss, adjust the parameters of the training set using fivefold cross validation. By reasonably adjusting the parameter β, the classification loss is made to be at a low value at the early stage of training to make the semantic segmentation model converge faster. If using a too low β value, it leads to the loss of noise from classification at the early stage of training, and a too high β value may lead to gradient explosion.

### Experimental parameter setting

The experiments selected Intel^®^ Core™ i7-10600 CPU with 2.90 GHz and NVIDIA GeForce RTX3090 GPU with 24 GB video memory. The experiment used PyTorch as the deep learning framework, dividing the training and testing sets into multiple batches, traversing all batches, and completing one iteration. The optimizer is selected as Adam, which automatically adjusts the learning rate.

### Evaluation indices

The classification task uses accuracy (ACC) as an evaluation index to quantify the ability to classify the degree of lodging. The segmentation task uses Precision, Recall, F1, and IoU indices to evaluate the model performance. The Precision refers to the proportion of predicted lodging area to actual lodging area; Recall represents the proportion of predicted lodging area to actual lodging area; F1 is the harmonic mean of precision and recall; and the IoU index is the overlap rate between the predicted area and actual area of lodging. The calculation formula is shown in Equations 2–6:


(2) $$ACC=\frac{T}{T+F}$$

(3) $$Pre=\frac{TP}{TP+FP}$$

(4) $$Rec=\frac{TP}{TP+FN}$$

(5) $$F1=2\times \frac{Pre\times Rec}{Pre+Rec}$$

(6) $$IoU=\frac{TP}{FN+TP+FP}$$

where TP is the number of pixels correctly identified as lodging wheat, TN is the number of pixels correctly identified as non-lodging wheat, FP is the number of pixels mistakenly identified as non-lodging wheat, FN is the number of pixels incorrectly identified as lodging wheat, T is the number of accurate classification plots, and F is the number of incorrectly classification plots.

## Results and discussion

### Training results

**Figure 11:**
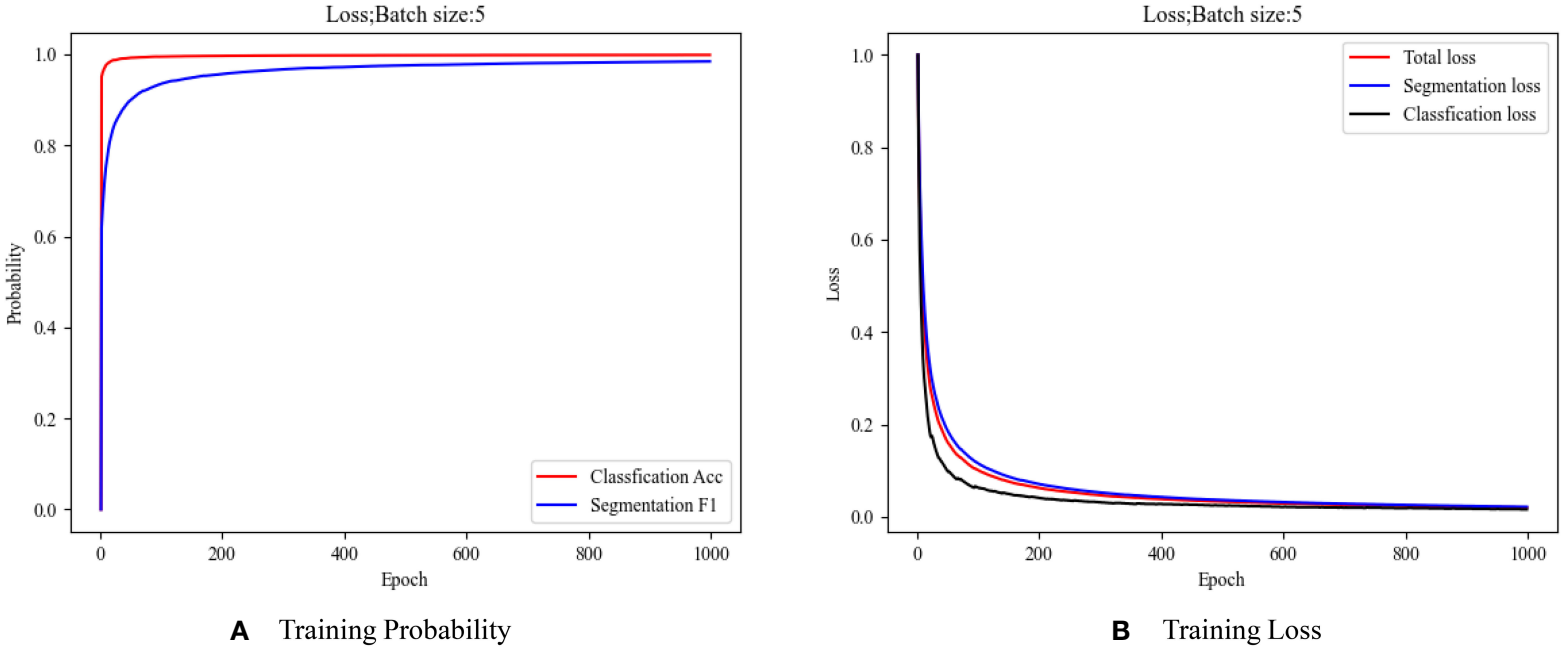
Model training index. **(A)** Training probability; **(B)** Training loss.

The accuracies of the training process, loss values, and F1 are shown in **Figure 11A**. Even if we weight the loss so that the semantic segmentation loss value accounts for a large proportion of the total loss value of the model, the accuracy is higher due to the fewer classification tasks, making the curve grow faster. It can be seen from **Figure 11B** that with the increase of iteration times, the overall trend of the loss function is smooth, and the convergence speed is fast, allowing multitask model training to be conducted.

### Quantitative analysis

According to **Tables 2**, **3**, the MLP_U-Net model used in this study was compared with various other models, including SegNet (area extraction task) (Badrinarayanan et al., 2017), U-Net (area extraction task), DeepLabV3 (area extraction task), U-Net++ (area extraction task), ResNet50 (classification task) (He et al., 2016), MobileNetv3 (classification task) (Howard et al., 2019), and conventional machine learning methods (Zhao et al., 2021). Using models from the previous research work, we developed traditional machine learning methods to extract wheat lodging areas for flyover 1, with an extraction error of 26.16%. Our model outperformed the traditional machine learning methods that relied on manual extraction of color features, which could not adapt to target size and complex background changes and had many false positives and missed detection areas. We compared flyover 1 with flyover 2 using a deep learning model. From the results, MLP_U-Net outperforms conventional machine learning methods and has higher model parameters and actual parameters in the deep learning models, which could accurately and efficiently complete the grading task of lodging degree and lodging area. The grading accuracy of lodging degrees in flyover 1 and flyover 2 reached 96.1% and 84.1%, respectively, and their F1 reached 81.3% and 82.0%, respectively. Moreover, the grading accuracy of lodging area reached 92.2% and 84.7%. Due to the deviation between the evaluation index of the dataset model and the actual index, the evaluation index of the flyover 1 model is low, while the actual index is high. Contrarily, flyover 2 has too many full negative samples, leading to the low calculation value of the confusion matrix.

**Table 2:** Evaluation index for dataset 1.

Models	Grading ACC of lodging degree (%)	Extraction of lodging area (%)				Grading ACC of lodging area (%)
		Pre	Rec	F1	IoU	
MLP_U-Net	96.1	78.5	84.2	81.3	68.5	92.2
SegNet	/	58.6	80.5	67.8	51.3	66.9
U-Net	/	64.2	84.7	73.0	57.5	88.3
DeepLabV3	/	74.9	83.9	79.1	65.5	81.8
U-Net++	/	80.3	78.8	79.6	66.1	78.6
ResNet50	88.3	/	/	/	/	/
MobileNetv3	91.6	/	/	/	/	/

**Table 3:** Evaluation index for dataset 2.

Models	Grading ACC of lodging degree (%)	Extraction of lodging area (%)				Grading ACC of lodging area (%)
		Pre	Rec	F1	IoU	
MLP_U-Net	84.1	89.0	76.0	82.0	69.5	84.7
SegNet	/	84.1	74.0	78.7	64.9	47.3
U-Net	/	75.5	82.6	78.9	65.1	80.9
DeepLabV3	/	89.6	76.9	82.7	70.6	81.7
U-Net++	/	90.3	71.2	79.7	66.2	71.0
ResNet50	77.9	/	/	/	/	/
MobileNetv3	83.6	/	/	/	/	/

### Ablation study

The ablation experiment aims to remove some parts of the network and identify whether network performance fluctuates by controlling variables (Ranchordas and Araújo, 2009). As a result, we performed ablation experiments on different modules to verify the effectiveness of MLP_U-Net. The goal was to test the module’s effectiveness and determine if the model had redundant modules. We removed the MLP structure module and channel attention mechanism module to verify the effectiveness of each module using the method of controlling variables. **Tables 4**, **5** show the results. Adding the ablated MLP module impacts the challenging dataset significantly, improving the grading ACC of the lodging area by 6.1% on flyover 2. The results indicate that adding the ablation channel attention mechanism impacts the simple dataset significantly, and the grading ACC of lodging degree and area improves by 0.6% and 1.3%, respectively, on flyover 1. Our model has a more robust recognition performance based on the simultaneous addition of both modules.

**Table 4:** Ablation study of dataset 1.

MLP_U-Net	Grading ACC of lodging degree (%)	Extraction of lodging area (%)				Grading ACC of lodging area (%)
		Pre	Rec	F1	IoU	
Ablation channel attention mechanism + Ablation MLP module	96.8	79.3	84.3	81.7	69.1	85.1
Ablation channel attention mechanism	95.5	82.2	82.3	82.2	69.8	90.9
Ablation MLP module	96.1	86.2	78.3	82.0	69.5	92.9

**Table 5:** Ablation study of dataset 2.

MLP_U-Net	Grading ACC of lodging degree (%)	Extraction of lodging area (%)				Grading ACC of lodging area (%)
		Pre	Rec	F1	IoU	
Ablation channel attention mechanism + Ablation MLP module	85.6	90.2	89.7	74.2	81.2	72.5
Ablation channel attention mechanism	86.4	90.5	73.7	81.2	68.4	80.9
Ablation MLP module	85.6	90.0	71.8	79.9	66.5	78.6

### Qualitative analysis

According to the regional trials of China’s national wheat varieties, the degree of wheat lodging is classified into five grades based on the Ministry of Agriculture and Rural Affairs of the People’s Republic of China’s (2007). The five grades are no lodging (grade 1) and slight lodging, with plant inclination angle less than or equal to 30° (grade 2). Other gradings are moderate lodging, with a plant inclination angle of 30°–45° (grade 3). Grade 4 reflects heavy lodging, with a plant inclination angle of 45°–60°, and grade 5 shows severe lodging, with a plant inclination angle of 60° or more. Due to the limited number of lodging plots in grades 2 and 4 in this study, grades 2 and 3 are collectively referred to as grade 3. Moreover, grades 4 and 5 are called grade 5 during data processing. **Figure 4** depicts the experimental results of flyover 1, where the blue area represents grade 1, the yellow area represents grade 3, and the red area represents grade 5. This grading shows that the number of correctly identified plots is 147, and the number of incorrectly identified plots is 7. **Figure 5** presents the experimental results of flyover 2, where the blue area represents grade 1, the orange area represents grade 2, the yellow area represents grade 3, and the red area represents grade 4. This study identified 116 plots correctly, and the number of plots incorrectly identified was 15.

This study also classified the lodging area of wheat into five grades: no lodging (grade 1) and slight lodging, with lodging area less than or equal to 30% (grade 2). Others are heavy lodging, with lodging areas between 30% and 60% (grade 3), and severe lodging, with lodging areas greater than or equal to 60% (grade 4). **Figure 6** shows the experimental results of flyover 1, depicting the number of correctly identified plots as 140 and the number of incorrectly identified plots as 14. **Figure 7** presents the experimental results of flyover 2, in which the number of plots correctly identified is 114 and the number of plots incorrectly identified is 17.

Our study used deep learning and image processing to significantly reduce the workload of manual statistics for wheat lodging detection when classifying large-scale wheat lodging areas under field conditions. Regarding subjectivity, the deep learning technique solves the problem of phenotypic errors caused by individual subjective differences techniques. As a result, the deep learning method is more stable in repeated measurements and is of great importance in wheat lodging detection. **Figures 5**, **7** show that several factors may impact the model’s accuracy. These factors are differences in lodging phenotypic characteristics among different wheat varieties, variable lighting conditions, different angles of UAV during image capture, different flight altitudes, and differences in geomorphic characteristics at different time sequences (Hasan et al., 2018; Madec et al., 2019; David et al., 2020). The problem of overlapping plot lodging areas still exists due to the vast lodging areas in flyover 2, indicating that the lodging wheat areas cover non-lodging wheat areas, resulting in grading errors.

### Analysis of data difference

As shown in **Table 6**, grade 1 accuracy of lodging detection is the highest owing to its low misclassification rate. However, most models are robust in distinguishing between lodging and non-lodging. Grades 2 and 3’s lodging detection accuracy is still low due to threshold problems causing misclassification of the lodging area. **Table 7** shows a significant difference in sample distribution between flyover 1 and flyover 2 due to temporal differences. For example, flyover 1 has a small number of lodging plots, while flyover 2 has the opposite trend. Different network models exhibit strong tendencies due to data differences. For instance, the network model of U-Net is shallower and tends to have positive samples, while U-Net++ balances features of different layers and has negative samples. MLP_U-Net has added suitable noise to the network, enhancing its adaptability and choosing appropriate network depth, refinement module, and adversarial nature of the multitasking. Since the lodging boundary of grades 2 and 3 is fixed, some values with minor area identification deviations may exceed the upper and lower thresholds, resulting in grading errors. Despite obtaining better performance, our model is poor in discriminating grades 2 and 3 lodgings with fewer samples and apparent thresholds. This model also performs poorly in a single dataset. It also has the disadvantage of an advantage interval. Suppose uniform training and testing are conducted using data from the same height and lodging area in the same plot. In that case, MLP_U-Net has no significant advantage over other models with a single task.

**Table 6:** Exploring the efficiency of lodging grading recognition.

ACC (%)	Grade	MLP_U-Net		SegNet		U-Net		DeepLabV3		U-Net++		ResNet50		MoblieNetv3	
		Dataset1	Dataset 2	Dataset 1	Dataset 2	Dataset 1	Dataset 2	Dataset 1	Dataset 2	Dataset 1	Dataset 2	Dataset 1	Dataset 2	Dataset 1	Dataset 2
Lodging degree	1	100	93.8	/								90.4	91.7	96.0	95.8
	3	69.2	71.0									53.8	41.9	53.8	67.7
	5	87.5	90.4									100	86.5	87.5	82.7
Lodging area	1	99.2	100	98.7	100	100	100	99.1	95.1	99.0	100	/			
	2	58.8	62.2	22.4	34.4	13.3	36.8	28.6	55.6	28.2	45.1				
	3	62.5	83.3	58.3	52.6	30.8	59.2	45.5	82.4	57.1	70.6				
	4	87.5	94.7	100	31.6	85.7	100	85.7	100	85.7	100				

**Table 7:** Distribution of testset sample data.

Dataset	Grade of lodging degree			Grade of lodging area			
	Grade 1	Grade 3	Grade 5	Grade 1	Grade 2	Grade 3	Grade 4
Dataset 1	125	13	16	124	13	9	8
Dataset 2	48	31	52	27	47	37	20

## Conclusion

This study suggests a novel approach to determine the grading system’s lodging level using deep learning-based image grading. Field experiments were conducted to validate the feasibility of the proposed method, and the results showed promising outcomes. We demonstrate that deep learning can be used to automatically calculate lodging degree and area, which can help assess the risk of large-area lodging yield reduction. Therefore, we constructed a new dataset comprising 82 winter wheat varieties of two-time series. We also proposed a detection method for winter wheat lodging grading, which could process the stitched images of UAVs with different flight heights, difficulties, time series, and plot sizes. Our study shows that the training and testing task can be completed through the single image of a small sample. We constructed a multitask neural network model MLP_U-Net for the plot to improve the generalization of small-sample data. This model aims to achieve segmentation and classification tasks for wheat lodging degrees and lodging areas. The improved shift MLP module structure is fused with the U-shaped structure by controlling the parameter quality and refining the features to develop the MLP_U-Net. Two tasks can add noise to each other and minimize the logical mismatch between the lodging degree and lodging area using a multitasking model. This study chose to weigh the loss to control the noise level and adjusted the weights of the two tasks at different training periods to prevent gradient explosion. Our goal is to maintain the effect of noise on model training by adding an appropriate amount of noise. We also compared various single-task models. Our results indicate that MLP_U-Net has high accuracy when UAV flight height is 30 m. Moreover, the accuracies of winter wheat lodging degree and lodging area grading are 96.1% and 92.2%, respectively, when the UAV flight height is 50 m. Winter wheat lodging degree and lodging area grading accuracies were 84.1% and 84.7%, respectively. By verifying lodging images of multiple wheat varieties through different parameters, MLP_U-Net can accurately and efficiently complete the task of lodging grading for winter wheat, which can meet the demand of high-throughput operation in the wheat field environment. These results can also provide technical support for determining lodging damage degree and damage assessment in the future. We aim to conduct grading of wheat lodging in future research by adopting a more reasonable grading model, combining lodging degree and lodging area to define lodging grading instead of “one size fits all” through thresholds. We also aim to construct a dataset with a large sample to balance the samples.

## Data availability statement

The raw data supporting the conclusions of this article will be made available by the authors, without undue reservation.

## Author contributions

HZ: Conceptualization, Software, Supervision, Writing – original draft, Writing – review & editing. XS: Formal analysis, Methodology, Writing – original draft. YW: Data curation, Writing – review & editing. GL: Writing – review & editing. JZ: Writing – review & editing. GZ: Funding acquisition, Project administration, Writing – review & editing. WH: Writing – original draft. HS: Methodology, Writing – review & editing.
